# Prognostic Value of Urinary Biomarkers in Proteinuria Progression in IgA Nephropathy Patients Treated with Budesonide

**DOI:** 10.3390/medicina61050807

**Published:** 2025-04-26

**Authors:** Christodoulos Keskinis, Eleni Moysidou, Stamatia Stai, Michalis Christodoulou, Georgios Lioulios, Sotirios-Spyridon Vamvakas, Maria Stella Trivyza, Panagiotis Pateinakis, Marios Papasotiriou, Maria Stangou

**Affiliations:** 1 School of Medicine, Aristotle University of Thessaloniki (AUTH), 54642 Thessaloniki, Greece; moysidoueleni@yahoo.com (E.M.); staimatina@yahoo.gr (S.S.); michalischristodoulou22@gmail.com (M.C.); pter43@yahoo.gr (G.L.); mstangou@auth.gr (M.S.); 2 Department of Nephrology, Papageorgiou Hospital, 56429 Thessaloniki, Greece; pateinakis@hotmail.com; 3 1st Department of Nephrology AUTH, Hippokration Hospital, 54642 Thessaloniki, Greece; 4 Department of Nutritional Science and Dietetics, University of Peloponnese, 24100 Kalamata, Greece; svamvakas@go.uop.gr; 5 Department of Nephrology and Renal Transplantation, University Hospital of Patras, 26504 Patras, Greece; mariastella421@gmail.com (M.S.T.); mpapasotiriou@yahoo.com (M.P.)

**Keywords:** IgA nephropathy, targeted-release budesonide, urinary biomarkers, Monocyte Chemotactic Protein-1 (MCP-1), Matrix MetalloProteinase-9 (MMP-9), clusterin (CLU)

## Abstract

***Background* & *Objectives*:** Targeted-release budesonide (TRB) is the first approved agent aimed at targeting the early pathogenetic cascade in IgA nephropathy (IgAN). ***Materials and Methods*:** This prospective study included Caucasian IgAN patients diagnosed within the last 5 years, who had started a 10-month TRB treatment and were followed in the outpatient clinic. All participants had been on the maximal supportive care dose for at least the previous 6 months. Kidney function and proteinuria levels were recorded at the start of TRB treatment (T0) and at 3, 6, and 10 months (T3, T6, and T10, respectively), while urinary monocyte chemotactic protein-1 (MCP-1), matrix metalloproteinase-9 (MMP-9) and clusterin (CLU) levels were measured at T0 and T3. ***Results*:** In the cohort of all patients (mean age 53.24 ± 12.76 years, estimated glomerular filtration rate (eGFR 52.84 ± 25.93 mL/min/1.73 m^2^, proteinuria 2.84 ± 1.26 g/24 h), significant correlations were observed at T0 between MMP-9 and MCP-1 (r = 0.74, *p* = 0.004), MMP-9 and uCLU (r = 0.77, *p* = 0.002), and MCP-1 and uCLU (r = 0.65, *p* = 0.01). At T3, a significant correlation between MMP-9 and urinary CLU (uCLU) persisted (r = 0.71, *p* = 0.03). Higher MCP-1 (r = −0.560, *p* = 0.046) and MMP-9 (r = −0.330, *p* = 0.012) levels at T0 were associated with reduced proteinuria. Conversely, increased clusterin at T3 (r = 0.599, *p* = 0.031) was associated with worsening proteinuria. ***Conclusions*:** The treatment response to TRB was heterogeneous, with recent diagnosis (RD) patients showing improved kidney function and proteinuria, while older diagnosis (OD) patients exhibited worsening biomarkers and declining kidney function. Therefore, early interventions are crucial in IgAN patients. Finally, the biomarkers studied can be used prognostically to monitor disease progression.

## 1. Introduction

Immunoglobulin A (IgA) nephropathy constitutes the most common primary glomerulonephritis worldwide [1,2,3,4,5]. A significant proportion of patients (20–25%) will require renal replacement therapy within 20–25 years of diagnosis, with the majority being young adults, typically diagnosed between 20 and 30 years of age [1,2,3,4]. This combination of early onset and poor renal prognosis underscores the need for effective treatments, as well as reliable diagnostic and prognostic biomarkers [6]. The disease can be diagnosed only with kidney biopsy, where mesangial hypercellularity is typically observed [7]. The pathogenesis of IgAN is complex, with many underlying immunological mechanisms that are still not fully understood [8,9,10,11]. One widely accepted model to describe this process is the “four-hit” hypothesis, which links the disease to the immune system in the mucosal membranes of the gastrointestinal and respiratory tracts [9]. In these mucosal sites, B lymphocytes are normally activated to protect against pathogens by producing IgA [12,13]. This activation is often triggered by antigens from the intestinal microbiome, which appear to initiate an inflammatory cascade [14,15,16,17,18,19]. Consequently, inhibiting this specific immunological activation in the intestinal flora is targeted through a new therapeutic formulation, involving the administration of TRB, which acts on the Peyer’s patches [2,12,20,21]. Two clinical studies (Nefigan and Nefigard) demonstrated that TRB significantly reduces proteinuria and maintains the GFR, confirming the initial promising findings published by Smerud et al. in 2011 [22,23,24]. This regimen is the first to receive official approval from the Food and Drug Administration (FDA) for the treatment of IgAN [2].

Biomarkers have been essential in risk stratification, diagnosis and treatment follow-up for many diseases [25,26,27]. The term “biomarker” was first introduced in 1989 as a “measurable and quantifiable biological parameter” [25]. A decade later, it was defined as a characteristic measured as an indicator of normal biological processes, pathogenic processes, or pharmacologic responses [26]. Currently, there are no validated diagnostic biomarkers for IgAN [7]. However, according to the KDIGO 2021 guidelines, the most reliable prognostic biomarkers are proteinuria and eGFR [7]. The measurement of deficiently galactosylated IgA1 (dg-IgA1) levels is not feasible in routine clinical practice, and its correlation with disease activity and prognosis remains a subject of ongoing debate. Identifying reliable prognostic biomarkers could play a crucial role in predicting disease progression and guiding therapeutic interventions [28,29,30,31,32,33,34].

MCP-1 is a crucial cytokine identified in humans, playing a key role in immune response [35,36,37,38,39]. It is produced by various cell types, with monocytes and macrophages being its primary sources. In the kidneys, MCP-1 is predominantly produced by tubular epithelial cells and is involved in interstitial inflammation and fibrosis, which are common processes in all renal diseases [36,37,38]. Elevated urinary MCP-1 levels have been identified as potential predictors of kidney fibrosis in conditions such as IgAN [39]. CLU, a 75 kDa glycoprotein, participates in various biological processes, including cell adhesion and apoptosis [40,41,42,43]. It is highly expressed in renal tubular epithelial cells following kidney injury and constitutes a major component of immune deposits in the kidney [42]. Due to its large molecular size, CLU is not filtered by the kidneys, making its urinary levels a specific marker for kidney damage [42]. Elevated uCLU levels have been observed following renal ischemia and nephrotoxin exposure, reflecting damage to both proximal and distal tubules [42]. Matrix metalloproteinases (MMPs), a family of enzymes that regulate extracellular matrix (ECM) turnover, are pivotal in various kidney diseases [44,45,46]. Among the 25 known MMPs, MMP-9 is particularly significant in kidney fibrosis [44,47,48]. It is produced by several renal cell types, including mesangial, glomerular, and epithelial cells, and is involved in ECM degradation [47,48]. MMP-9 levels are elevated in kidney diseases such as IgAN, lupus nephritis, and diabetic nephropathy, reflecting its role in renal injury and fibrosis [45,49]. The aforementioned urinary cytokines’ excretion can reflect tubular dysfunction and interstitial fibrosis, and their use as disease prognostic biomarkers have not been thoroughly investigated in IgAN. The objective of this study is to evaluate the clinical utility of specific urinary biomarkers, MCP-1, MMP-9 and clusterin, for monitoring disease progression and treatment response in IgAN patients. These biomarkers, which are associated with proximal tubular dysfunction and interstitial fibrosis, were analyzed in relation to proteinuria and eGFR. Furthermore, the study aims to determine whether baseline levels and early changes in these biomarkers can serve as prognostic indicators of therapeutic response to TRB. Given that the chronicity of histological lesions may affect both biomarker expression and treatment efficacy, patients were stratified into two subgroups based on the time interval since diagnosis. This stratification was designed to evaluate whether the timing of intervention influences biomarker dynamics and treatment outcomes. The innovation in this study is based on the combined monitoring of urinary biomarkers representing the set of pathophysiological processes of renal damage in IgAN (inflammation, tubular damage, interstitial fibrosis) in a group of patients who received specific treatment aimed at reducing the production of abnormally galactosylated IgA (budesonide) both at the beginning of treatment and during follow-up. In this way, it is possible to monitor not only the effect of treatment on the different pathways of kidney damage but also potentially detect biomarker signatures that could be predictive of response to treatment.

## 2. Materials and Methods

### 2.1. Study Design

This prospective study included IgAN patients diagnosed within the last 10 years who met specific inclusion criteria. All participants were treated with TRB at 16 mg daily for 9 months, followed by a 1-month taper. Regular follow-up assessments were conducted at the start of treatment (T0), at 3 months (T3), 6 months (T6), and 10 months (T10) after treatment initiation. The Chronic Kidney Disease Epidemiology Collaboration (CKD-EPI) equation was used to estimate eGFR, and proteinuria severity was evaluated through 24-h urinary protein (Uprot) levels and the urine protein-to-creatinine ratio (UPCR). The study was not designed with the methodology and criteria of a randomized controlled trial, but aims to correlate specific biomarkers measured at the start and at the three-month mark of treatment with budesonide with the actual data that emerge during the follow-up of these patients.

### 2.2. Inclusion and Exclusion Criteria

Inclusion Criteria:

Age 18 to 75 years

- Caucasian ethnicity
- Primary IgAN confirmed by kidney biopsy
- Diagnosis within the past 10 years
- eGFR > 30 mL/min/1.73 m^2^ (based on CKD-EPI equation)
- Uprot > 1 g/day in two consecutive measurements, at least one month apart
- Treatment with the maximal tolerated dose of angiotensin or aldosterone axis inhibitors and/or sodium-glucose co-transporter-2 (SGLT-2i) for at least 6 months prior to enrollment
- Signed informed consent

Exclusion Criteria:

- IgA vasculitis or secondary IgAN
- Diabetes mellitus
- Liver cirrhosis
- Use of steroids or immunosuppressive therapy within the last 6 months

### 2.3. Laboratory Analyses

ELISA was applied to evaluate urinary excretion of CLU, MCP-1 and MMP-9 at T0 and T3. MCP-1, MMP-9 and clusterin concentrations were measured using commercially available sandwich ELISA kits (MCP-1: DCP00, MMP-9: DMP900; Clusterin: DCLU00; R&D Systems, Minneapolis, MN, USA). The tests were performed according to the manufacturer’s recommended protocols.

### 2.4. Study Endpoints

**Figure 1:**
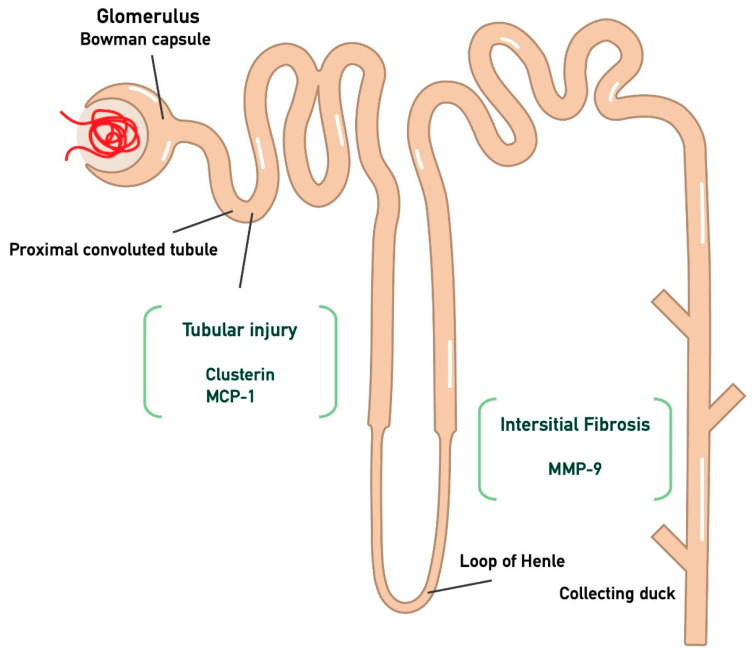
Kidney injury biomarkers associated with tubular atrophy and interstitial damage. In particular, CLU and MCP-1 are associated with tubular injury, while MMP-9 is linked to interstitial fibrosis. (Monocyte Chemotactic Protein-1 (MCP-1); Matrix MetalloProteinase-9 (MMP-9); clusterin (CLU)).

The primary endpoints of the study are the monitoring of proteinuria and eGFR at the time points T0, T3, T6, and T10 in the participants, in relation to the three biomarkers (MCP-1, MMP-9, Clusterin) measured at the start of treatment and at the three-month follow-up. In particular, MCP-1 and clusterin were chosen as markers to assess proximal tubular dysfunction, while interstitial fibrosis was evaluated through MMP-9, as illustrated in Figure 1. The aim of the study is to determine whether there is any correlation between these biomarkers, so that in the future they could be used prognostically to predict whether a patient is likely to respond to treatment with targeted-release budesonide. Furthermore, given that these biomarkers mainly represent tubular-interstitial atrophy and fibrosis, which are typically chronic histological lesions in IgAN, the decision was made to separate patients into two sub-populations. Specifically, patients were divided into two groups: those with a recent diagnosis (RD, *n* = 8, *t* < 6 months) and those with an old diagnosis (OD, *n* = 6, *t* > 6 months).

### 2.5. Statistical Analysis

The IBM SPSS 26.0 (SPSS Inc., Chicago, IL, USA) package was used for statistical analysis. The Shapiro–Wilk test was applied to estimate normality of all quantitative variables and, accordingly, mean and standard deviation or median value and interquartile range (IQR) were used to describe the results. The *t*-test for dependent variables, and Wilcoxon test were applied to compare the mean/median vales of two dependent groups, respectively, and the Spearman test was used to estimate correlation between two independent parameters. *p* values < 0.05 were considered statistically significant. All graphs were created using the GraphPad Prism9 program.

## 3. Results

### 3.1. Study Population

Fourteen patients with IgAN (11 males, 3 females; mean age 53.24 ± 12.76 years) were enrolled and followed for 10 months. All participants were on the maximum tolerated dose of antiproteinuric angiotensin–aldosterone axis inhibitors. The RD group included eight patients who were diagnosed on average 2.5 months (range: 2 to 3.75 months) prior to the study. In contrast, the OD group consisted of six patients who had been diagnosed on average 28.5 months (range: 9.25 to 42 months) prior to the study. A total of 5 patients were receiving SGLT2 inhibitors, specifically dapagliflozin 10 mg daily. Of these patients, 2 patients were in the RD group and 3 were in the OD group. Additionally, all patients in the OD group had previously received systemic corticosteroids. The demographic data of the participants at the initiation of treatment are summarized in Table 1 below.

### 3.2. Changes in Renal Function and Urinary Cytokines During TRB Treatment

In the whole cohort of patients, changes in eGFR and Uprot levels during TRB treatment and in urine CLU, MCP-1 and MMP-9 during the first 3 months are depicted in Table 2.

### 3.3. Biomarker Correlations in IgA Nephropathy: Inflammation, Fibrosis, and Treatment Response

In the entire cohort of IgAN patients, significant correlations were observed between various biomarkers and clinical parameters, providing insights into the potential associations between inflammation, fibrosis and kidney function in patients undergoing treatment with TRB. A significant positive correlation was found between MCP-1 at baseline (T0) and MMP-9 at baseline (T0) (r = 0.742, *p* = 0.004), suggesting that higher baseline levels of MCP-1, a marker of inflammation, are associated with increased levels of MMP-9, a marker of fibrosis. This indicates that inflammation may be linked to fibrosis at the onset of treatment. Additionally, a significant positive correlation was found between MCP-1 (T0) and uCLU (T0) (r = 0.65, *p* = 0.01), reflecting a relationship between inflammation and tubulointerstitial injury at T0. Moreover, a significant positive correlation between MMP-9 (T0) and uCLU (T0) (r = 0.77, *p* = 0.002) further supports the idea that inflammation and fibrosis are interconnected processes in the early stages of treatment. At the 3-month follow-up (T3), a significant positive correlation between MMP-9 (T3) and uCLU (T3) (r = 0.71, *p* = 0.03) further emphasizes the ongoing link between fibrosis and tubulointerstitial injury, indicating that both biomarkers may be valuable for monitoring the disease’s progression over time.

Given the exploratory nature and relatively small sample size of our study (*n* = 14), a post hoc power analysis was performed to estimate the statistical power of detecting meaningful changes in key outcomes. For the primary endpoint of 24-h proteinuria (Uprot), a significant reduction was observed from baseline (T0: 2.84 ± 1.26 g/day) to 10 months (T10: 1.73 ± 0.66 g/day). With a moderate within-subject correlation (r = 0.6), the effect size (Cohen’s d) was estimated at approximately 0.58, indicating a moderate effect. Using a two-tailed paired-sample *t*-test with a significance level of α = 0.05, the achieved power was calculated to be approximately 85%, suggesting that the study was sufficiently powered to detect this change in proteinuria. In contrast, changes in urinary biomarkers showed more subtle and highly variable results. Specifically, MCP-1 increased from 232.87 ± 238.57 to 262.69 ± 199.34 pg/mg creatinine, corresponding to a small effect size (d ≈ 0.13) and an estimated power of <20%. MMP-9 increased from a median of 0.15 (IQR: 0.04–0.95) at T0 to 0.22 (IQR: 0.10–0.89) at T3, and clusterin rose from 34.07 (IQR: 11.65–106.18) at T0 to 61.19 (IQR: 13.81–162.8) at T3, both demonstrating high inter-individual variability and non-parametric distributions.

Furthermore, a significant negative correlation was found between MCP-1 (T0) and urinary protein levels at T0 (r = −0.560, *p* = 0.046), suggesting that higher MCP-1 levels at baseline may be associated with lower proteinuria, potentially reflecting an early effect of inflammation on reducing protein excretion. At 3 months (T3), a significant positive correlation between clusterin (T3) and urinary protein levels (Uprot) (r = 0.599, *p* = 0.031) suggests that uCLU levels correlate with proteinuria. Finally, significant negative correlations were observed between MMP-9 (T0) and Uprot (T0) (r = −0.330, *p* = 0.012), suggesting that higher MMP-9 levels at T0 may be associated with reduced proteinuria, potentially reflecting the effects of fibrosis on kidney filtration. All the statistically significant findings are summarized in Table 3.

### 3.4. Comparison of Biomarkers and Clinical Parameters Between OD and RD Groups in IgA Nephropathy

**Figure 2:**
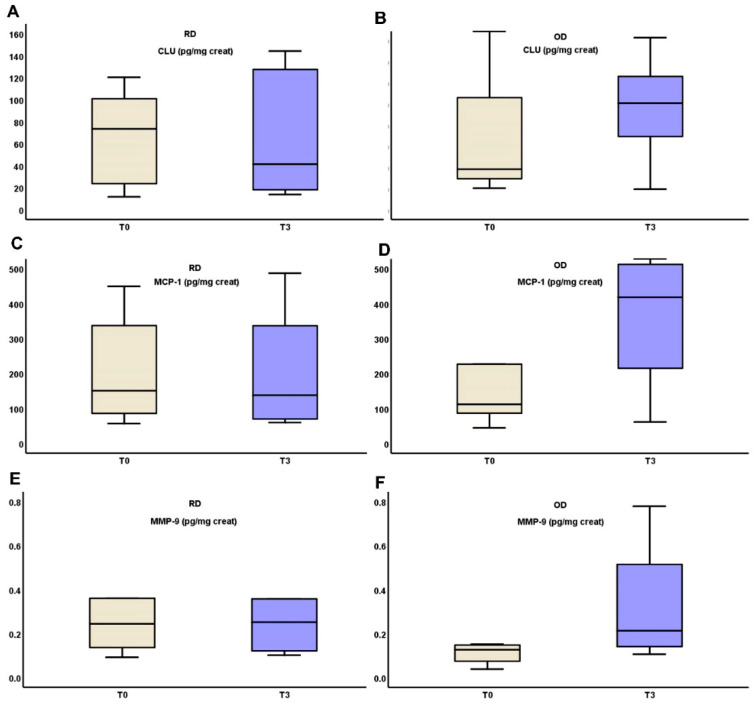
Changes in urinary excretion of clusterin (**A**,**B**), MCP-1 (**C**,**D**) and MMP-9 (**E**,**F**) three months after starting on TRB treatment in RD and OD IgAN patients, respectively (Monocyte Chemotactic Protein-1 (MCP-1); Matrix MetalloProteinase-9 (MMP-9); clusterin (CLU), targeted-release budesonide (TRB), recent diagnosis (RD), old diagnosis (OD), IgA nephropathy (IgAN)).

In the RD group, uCLU, MMP-9 and MCP-1 levels remained stable from T0 to T3 (90.87 (103.63–26.49) vs. 52.87 (131.43 vs. 19.5), *p* = 0.321, 0.36 (1.33–0.22) vs. 0.38 (16.62–0.19), *p* = 0.321 and 224.93 ± 151.79 vs. 222.51 ± 185.27, *p* = 0.423 respectively), while all three substances increased in the OD group from T0 to T3 (34.08 (124.46–18.44) vs. 85.3 (135.36–18.22), *p* = 0.306, 0.13 (19.6–0.78) vs. 0.22 (16.03–0.11), *p* = 0.306 and 99.61 (396.02–70.21) vs. 316.61 (527.34–73.58), *p* = 0.416, respectively). All of these biomarkers ‘alterations are depicted in Figure 2.

**Figure 3:**
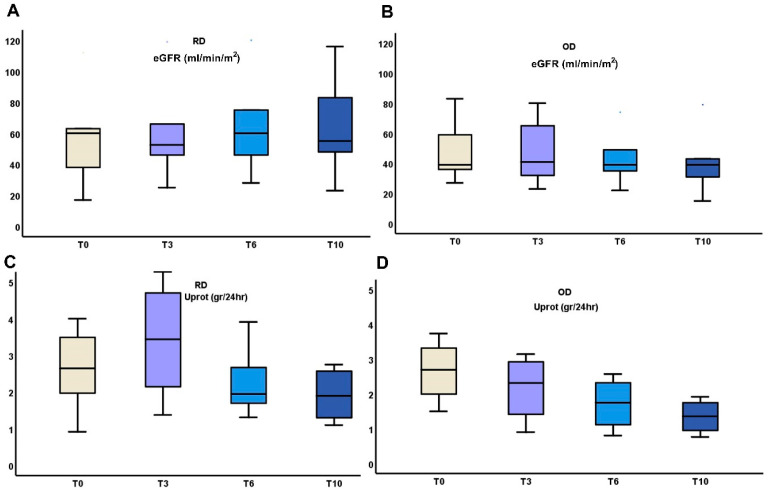
Changes in kidney function (**A**,**B**), and proteinuria (**C**,**D**) during the 10 months administration of TRB treatment in RD or OD IgAN patients (Monocyte Chemotactic Protein-1 (MCP-1); Matrix MetalloProteinase-9 (MMP-9); clusterin (CLU), Targeted-release budesonide (TRB), recent diagnosis (RD), old diagnosis (OD), IgA nephropathy (IgAN).

The above early changes in urinary cytokines were followed by differences in the outcome of eGFR and proteinuria between RD and OD patients (Table 2, Figure 3).

In the RD group, eGFR showed a slight improvement from 55.37 ± 29.11 mL/min/m^2^ at T0 to 64.14 ± 28.67 mL/min/m^2^ at 6 months, followed by a small decrease to 63.33 ± 32.22 mL/min/m^2^ at 10 months. Proteinuria decreased from 2.89 ± 1.5 g/24 h at T0 to 1.92 ± 0.68 g/24 h at 10 months. In contrast, the OD group experienced a decline in eGFR from 49.4 ± 21.71 mL/min/m^2^ at T0 to 41.4 ± 23.59 mL/min/m^2^ at 10 months. Proteinuria also decreased in this group from 2.78 ± 0.85 g/24 h at T0 to 1.35 ± 0.5 g/24 h at 10 months. While both groups showed reductions in proteinuria, the RD group exhibited more stable kidney function, whereas the OD group demonstrated a progressive decline in eGFR.

## 4. Discussion

In recent years, research interest in IgAN has increasingly focused on identifying prognostic and diagnostic biomarkers, especially in urine, as a means to aid early diagnosis, track disease progression, and assess the effectiveness of treatments [6]. The present study aimed to explore the potential utility of three urinary biomarkers, MCP-1, clusterin and MMP-9, as prognostic indicators in IgAN patients receiving TRB. These biomarkers are known to play pivotal roles in inflammation, fibrosis and tubular injury, processes that are central to the pathophysiology of IgAN [50,51,52]. The typical phenotype of IgAN is characterized by a slow but steadily progressive decline in kidney function [1,50,53]. The disease gradually leads to chronic histological changes, primarily tubular atrophy and interstitial fibrosis, which contribute to the progressive deterioration of renal function [50,51]. We also aimed to investigate the relationship between these biomarkers and clinical outcomes such as kidney function (measured by eGFR) and proteinuria.

Our findings suggest that urinary MCP-1, CLU and MMP-9 levels may serve as valuable prognostic biomarkers in IgAN. Notably, we observed significant correlations between the levels of these biomarkers, further supporting the idea that inflammation and fibrosis are closely intertwined in the early stages of IgAN, indicating that the disease is a chronic inflammatory condition that progressively leads to kidney function deterioration [54].

### 4.1. The Link Between MCP-1 and MMP-9

At treatment initiation, the strong positive correlation between MCP-1 and MMP-9 (r = 0.742, *p* = 0.004) is particularly significant. This correlation between MCP-1 and MMP-9 at T0 suggests that inflammation and fibrosis are closely intertwined at the onset of treatment. This is consistent with the hypothesis that ongoing inflammation promotes the development of fibrosis in the kidney, leading to progressive renal damage. The dual targeting of inflammation and fibrosis could therefore be a promising therapeutic strategy for IgAN, aiming to halt the progression of kidney damage by addressing both the inflammatory and fibrotic components of the disease simultaneously. A recently FDA-approved therapeutic regimen (sparsentan) may slow the disease progression and the aforementioned inflammatory response [55]. At the 3-month follow-up (T3), the correlation between MCP-1 and MMP-9 decreases (r = 0.527, *p* = 0.064) is not statistically significant. This suggests that, while the relationship between inflammation and fibrosis persists over time, TRB administration helps reduce the ongoing inflammation in the tubules and interstitium.

### 4.2. Clusterin and Its Correlation with MCP-1 and MMP-9

At treatment initiation (T0), the positive correlation between MCP-1 and urinary CLU (r = 0.65, *p* = 0.01) suggests a direct relationship between inflammation and tubulointerstitial injury. This finding highlights the central role of inflammation in the early stages of kidney injury in IgAN, supporting the concept that targeting inflammation early on could help prevent further kidney damage. Furthermore, the strong positive correlation between MMP-9 and uCLU at treatment initiation (r = 0.77, *p* = 0.002) further reinforces the link between fibrosis and tubulointerstitial injury at the onset of treatment. MMP-9, a matrix metalloproteinase involved in extracellular matrix remodeling, is closely associated with fibrosis, and its elevated levels, along with the concurrent rise in CLU, suggest that fibrotic changes contribute significantly to kidney damage at this stage. This finding underscores the interconnectedness of inflammation and fibrosis in the early phases of IgAN, highlighting the importance of targeting both pathways simultaneously for effective therapeutic intervention. On the other hand, at the 3-month follow-up (T3), the positive correlation between MMP-9 and uCLU (r = 0.71, *p* = 0.03) emphasizes the continuing interplay between fibrosis and tubulointerstitial injury over time. Even after the initial phase of treatment, fibrotic processes remain central to the ongoing kidney damage, with elevated levels of CLU indicating that fibrosis contributes to the persistence of kidney injury.

### 4.3. Correlation Between Biomarkers and Proteinuria in IgA Nephropathy

At treatment initiation (T0), a notable negative correlation was observed between MCP-1 and proteinuria (r = −0.560, *p* = 0.046). The negative correlation suggests that, at the start of treatment, higher levels of MCP-1 may be associated with a reduction in proteinuria. This relationship is likely complex and further studies would be needed to elucidate the precise mechanisms underlying this observation. It is also important to note that higher MCP-1 levels were associated with reduced proteinuria.

In contrast, the positive correlation between clusterin and urinary protein levels at 3 months (T3) (r = 0.599, *p* = 0.031) highlights the potential role of clusterin as a marker of ongoing kidney damage. Clusterin, a protein involved in cellular stress responses and tissue injury, has been shown to increase in the urine during episodes of tubulointerstitial injury. The positive correlation with proteinuria at 3 months suggests that, as kidney injury progresses and fibrosis continues to develop, clusterin levels increase, reflecting severe and sustained tubulointerstitial damage. Elevated clusterin levels at T3 may therefore correlate with worsening proteinuria, and its presence could be indicative of the persistence of kidney injury despite treatment. This suggests that clusterin might serve as a valuable biomarker for monitoring disease progression and the effectiveness of interventions, particularly in chronic or more advanced stages of IgAN.

Similarly, the correlation between MMP-9 and urinary protein levels provides additional insight into the dynamic processes underlying kidney injury in IgAN. At T0, a negative correlation was observed between MMP-9 and urinary protein levels (r = −0.330, *p* = 0.012). The negative correlation at T0 suggests that higher MMP-9 levels might be associated with a reduction in proteinuria. This finding could reflect an early compensatory response, where MMP-9 facilitates tissue repair and matrix remodeling, thereby helping to restore the integrity of the glomerular filtration barrier and reduce protein leakage into the urine. This might be particularly relevant in the initial stages of IgAN, when inflammatory processes are active and potentially reversible damage is being managed.

However, at 3 months (T3), the correlation between MMP-9 and proteinuria becomes weaker and it is not statistically significant (r = −0.220, *p* = 0.067). This finding suggests that, while MMP-9 continues to be involved in kidney repair processes, its role in reducing proteinuria may diminish as the disease progresses. By this point, fibrosis may have become a more dominant feature of the disease, limiting the capacity for repair and exacerbating kidney injury. The weaker correlation at T3 could therefore indicate that MMP-9′s ability to mitigate proteinuria diminishes over time as fibrotic changes become more pronounced, highlighting the shift from inflammation-driven to fibrosis-driven pathology in chronic kidney disease.

Together, these findings underscore the complex relationship between inflammation, fibrosis, and kidney function in IgAN. The negative correlations between MCP-1 and MMP-9 with proteinuria at treatment initiation suggest that inflammation and tissue repair processes may have an early protective role in reducing protein leakage. However, as the disease progresses, the positive correlation between clusterin and proteinuria at 3 months indicates that ongoing tubulointerstitial injury and fibrosis may lead to worsening kidney function. The near-significant negative correlation between MMP-9 and proteinuria at T3 further supports the notion that fibrotic processes, rather than inflammation, become the dominant force driving kidney injury and proteinuria over time.

These results highlight the potential for using biomarkers like MCP-1, MMP-9 and clusterin to monitor disease progression and therapeutic response in IgAN. While targeting inflammation and fibrosis early in the disease may help to reduce proteinuria and prevent further kidney damage, the shift toward fibrosis-driven kidney injury suggests that interventions aimed at modulating fibrosis may be required in later stages of the disease. Further research is needed to better understand the temporal dynamics of these biomarkers and their utility in guiding clinical decision-making for IgAN patients.

### 4.4. Comparison Between RD and OD Groups

These results highlight the importance of early intervention in IgAN. Patients with RD appear to benefit more from TRB treatment, with better preservation of kidney function compared to those with OD. This suggests that the degree of renal fibrosis and inflammation at the time of treatment initiation may impact the response to therapy. Additionally, the stable levels of MCP-1 and MMP-9 in the RD group, as compared to the increases observed in the OD group, further support the idea that inflammation and fibrosis play a significant role in disease progression and may affect treatment outcomes. In fact, TRB constitutes an immunosuppressive treatment. As a result, nephrons exhibiting immunologically active inflammation are expected to benefit the most [50,51]. IgAN patients who have recently been diagnosed are likely to have active inflammation, making them prime candidates for targeted therapeutic interventions aimed at modulating the immune response and preventing further renal damage [52]. The need to differentiate between immunologically active disease and proteinuria resulting from chronic damage due to other comorbidities is crucial in the management of IgAN [50,51]. A key criterion for detecting potential immunological activity in patients with long-standing IgAN, who may require immunosuppressive therapy, is the performance of repeat kidney biopsies [50]. This approach allows for histological classification using the activity and chronicity criteria applied in systemic lupus erythematosus nephritis [50,56]. By distinguishing between active inflammation and irreversible damage, clinicians can better tailor treatment strategies, ensuring that patients with ongoing immunological activity receive the appropriate interventions to prevent further renal deterioration.

### 4.5. Implications for Clinical Practice

The findings from our study have several important implications for clinical practice. First, they underscore the potential utility of the studied biomarkers in monitoring disease progression and predicting treatment response in IgAN. These biomarkers may complement existing clinical parameters, such as eGFR and proteinuria, providing a more comprehensive assessment of disease activity. Given that proteinuria and eGFR are the most widely used prognostic markers for IgAN, adding urinary biomarkers to the diagnostic and monitoring toolkit could improve risk stratification and help guide treatment decisions.

This is one of the few studies that presents data on urinary biomarker excretion in IgAN patients undergoing TRB treatment. Overall data are presented on the effect of TRB administration on inflammation (MCP-1), tubular epithelial damage (clusterin), and induced kidney fibrosis (MMP-9). In addition, the inclusion of patients with a recent and older history of IgAN who have received a full course of TRB treatment and show a different biomarker excretion profile may help to discriminate patients with specific characteristics that indicate adequate or no response to treatment and remission of the disease. This distinction is particularly important in recognizing patients with active histological lesions, as they are more likely to benefit from intervention, in contrast to those with advanced chronic kidney disease who may have a less favorable response to treatment.

### 4.6. Limitations

Although the biomarkers MCP-1, MMP-9, and clusterin showed trends suggesting potential associations with disease progression, their role as predictors of treatment response remains uncertain and requires further investigation in larger, more powerful studies. One important limitation of this study is the lack of adjustment for multiple comparisons. Although the statistical analyses were hypothesis-driven and based on predefined biomarker–parameter relationships, the relatively small sample size and the number of pairwise tests conducted may increase the risk of Type I error. As a result, the observed associations, especially those with borderline significance, should be interpreted with caution. These findings should be considered exploratory and require validation in future studies with larger populations, where formal correction methods such as Bonferroni or false discovery rate (FDR) can be appropriately applied.

Furthermore, our analysis also revealed that the study was underpowered to detect small-to-moderate changes in urinary biomarkers due to high inter-individual variability and the small sample size. For example, MCP-1 showed only a small effect size (d ≈ 0.13), and the estimated power for detecting changes in MCP-1 was less than 20%. Similarly, MMP-9 and clusterin demonstrated low statistical power (estimated power < 30%) to detect meaningful changes due to their non-parametric distributions and high variability across participants. These findings emphasize the need for caution when interpreting biomarker-related outcomes in this study, particularly given the small sample size and the exploratory nature of the analysis. Based on the aforementioned findings, the estimated power for detecting significant changes in MMP-9 and clusterin was < 30%. These findings confirm that, while the study was adequately powered to detect changes in proteinuria, it was not sufficiently powered to robustly assess small-to-moderate changes in urinary biomarkers. Consequently, the biomarker-related findings should be interpreted with caution. Further validation in larger, adequately powered cohorts is essential to confirm these preliminary observations and more accurately assess the prognostic utility of MCP-1, MMP-9, and clusterin as reliable indicators of therapeutic response, particularly in the context of targeted treatments such as TRB. Specifically, our team continues to recruit IgAN patients undergoing TRB treatment, and additional measurements of various biomarkers will be taken at multiple time points in the future to evaluate their prognostic value in a broader patient population.

While our study provides valuable insights into the potential role of urinary biomarkers in IgAN, it is not without limitations. The sample size was relatively small (14 patients), and the study was conducted over a relatively short period (10 months). Larger, multi-center studies are needed to validate our findings and assess the long-term prognostic value of MCP-1, CLU, and MMP-9 in IgAN. Furthermore, additional biomarkers, including those related to the immune system and kidney injury, may provide further insights into disease progression and treatment response. Moreover, future studies should explore the relationships between these biomarkers and responses to other up-coming novel therapies.

## 5. Conclusions

Our study provides preliminary insights into the potential role of urinary biomarkers, such as MCP-1, clusterin and MMP-9, in monitoring disease progression and treatment response in IgAN. These biomarkers appear to reflect critical processes associated with inflammation, fibrosis, and tubulointerstitial injury. The observed correlation between MCP-1 and MMP-9 at treatment initiation may suggest that targeting both inflammatory and fibrotic pathways could be a promising approach, especially in the early stages of the disease. Clusterin and MMP-9 may also offer value in tracking ongoing renal damage. However, given the small sample size of this study, the results should be interpreted with caution. Larger, well-designed studies are required to further investigate these findings and determine the clinical relevance of these biomarkers for improving patient management and therapeutic strategies in IgAN.

## Figures and Tables

**Table 1:** Demographic data of all 14 participants at treatment initiation (T0) (Male (M), (female (F)), sodium-glucose co-transporter-2 inhibitor (SGLT-2i), estimated glomerular filtration rate (eGFR), proteinuria (Uprot).

Clinical Parameters at Treatment Initiation (T0)	All Patients
N	14
M/F	11/3
Age (years)	53.24 ± 12.76
Systolic blood pressure (mmHg)	129.29 ± 9.38
Diastolic blood pressure (mmHg)	75.71 ± 11.58
Time since diagnosis (months)	4 (2–26.25)
Previous steroid treatment, *n* (%)	6 (42.86%)
SGLT-2i treatment, *n* (%)	5 (35.71%)
eGFR (mL/min/1.73 m^2^)	52.84 ± 25.93
Uprot (g/24h)	2.84 ± 1.26

**Table 2:** Changes in urinary biomarkers, eGFR and proteinuria in the whole cohort, RD and OD IgAN patients after starting on TRB treatment (estimated glomerular filtration rate (eGFR), recent diagnosis (RD), old diagnosis (OD), Monocyte Chemotactic Protein-1 (MCP-1), Matrix MetalloProteinase-9 (MMP-9), clusterin: (CLU).

	T0	T3	T6	T10
All Patients				
CLU (pg/mg creat)	34.07 (11.65–106.18)	61.19 (13.81–162.8)		
MCP-1 (pg/mg creat)	232.87 ± 238.57	262.69 ± 199.34		
MMP-9 (pg/mg creat)	0.15 (0.04–0.95)	0.22 (0.1–0.89)		
eGFR (mL/min/m^2^)	52.84 ± 25.93	55.92 ± 26.35	55.66 ± 26.37	53.37 ± 29.55
Uprot (g/24 h)	2.84 ± 1.26	3 ± 1.45	2.06 ± 0.81	1.73 ± 0.66
RD				
CLU (pg/mg creat)	90.87 (103.63–26.49)	52.87 (131.43–19.5)		
MCP-1 (pg/mg creat)	224.93 ± 151.79	222.51 ± 185.27		
MMP-9 (pg/mg creat)	0.36 (1.33–0.22)	0.38 (16.62–0.19)		
eGFR (mL/min/m^2^)	55.37 ± 29.11	55.88 ± 29.05	64.14 ± 28.67	63.33 ± 32.22
Uprot (g/24 h)	2.89 ± 1.5	3.43 ± 1.52	2.23 ± 0.83	1.92 ± 0.68
OD				
CLU (pg/mg creat)	34.08 (124.46–18.44)	85.3 (135.36–18.22)		
MCP-1 (pg/mg creat)	99.61 (396.02–70.21)	316.61 (527.3–73.6)		
MMP-9 (pg/mg creat)	0.13 (19.6–0.78)	0.22 (16.03–0.11)		
eGFR (mL/min/m^2^)	49.4 ± 21.71	48.2 ± 23.68	43.8 ± 19.46	41.4 ± 23.59
Uprot (g/24 h)	2.78 ± 0.85	2.17 ± 0.97	1.72 ± 0.76	1.35 ± 0.5

**Table 3:** Significant correlations between inflammatory, fibrotic markers and proteinuria in IgA nephropathy patients undergoing TRB (targeted-release budesonide (TRB), Monocyte Chemotactic Protein-1 (MCP-1); Matrix MetalloProteinase-9 (MMP-9); clusterin (CLU)).

Biomarker Pair	Correlation Coefficient (r)	*p*-Value	Interpretation
MCP-1 (T0) & MMP-9 (T0)	0.742	0.004	Positive correlation between MCP-1 (inflammation) and MMP-9 (fibrosis), indicating a link between inflammation and fibrosis at treatment initiation.
MCP-1 (T0) & uCLU (T0)	0.65	0.01	Positive correlation between MCP-1 (inflammation) and uCLU (tubulointerstitial injury), suggesting a relationship between inflammation and kidney injury.
MMP-9 (T0) & uCLU (T0)	0.77	0.002	Strong positive correlation between MMP-9 (fibrosis) and uCLU (tubulointerstitial injury), further supporting the connection between inflammation and fibrosis at treatment onset.
MMP-9 (T3) & uCLU (T3)	0.71	0.03	Positive correlation between MMP-9 and uCLU at 3 months, emphasizing the continued link between fibrosis and tubulointerstitial injury over time.
MCP-1 (T0) & Uprot (T0)	−0.560	0.046	Negative correlation between MCP-1 and urinary protein levels at baseline, suggesting that higher MCP-1 levels at the start of treatment may be associated with reduced proteinuria.
Clusterin (T3) & Uprot (T3)	0.599	0.031	Positive correlation between urinary clusterin and proteinuria at 3 months, suggesting that clusterin levels correlate with protein excretion.
MMP-9 (T0) & Uprot (T0)	−0.330	0.012	Negative correlation between MMP-9 and proteinuria at baseline, suggesting that higher MMP-9 levels may be associated with reduced proteinuria at the start of treatment.

## Data Availability

All data are available upon request from the corresponding author.
